# Up-regulation of *miR-95-3p* in hepatocellular carcinoma promotes tumorigenesis by targeting p21 expression

**DOI:** 10.1038/srep34034

**Published:** 2016-10-04

**Authors:** Jian Ye, Yufeng Yao, Qixue Song, Sisi Li, Zhenkun Hu, Yubing Yu, Changqing Hu, Xingwen Da, Hui Li, Qiuyun Chen, Qing K. Wang

**Affiliations:** 1 Key Laboratory of Molecular Biophysics of the Ministry of Education, College of Life Science and Technology and Center for Human Genome Research, Huazhong University of Science and Technology, Wuhan, P. R. China; 2 Center for Cardiovascular Genetics, Department of Molecular Cardiology, Cleveland Clinic; Department of Molecular Medicine/CCLCM, Department of Genetics and Genome Sciences, Case Western Reserve University, Cleveland, OH 44195, USA

## Abstract

Hepatocellular carcinoma (HCC) is one of the most common malignant cancers. To elucidate new regulatory mechanisms for heptocarcinogenesis, we investigated the regulation of p21, a cyclin-dependent kinase (CDK) inhibitor encoded by *CDKN1A*, in HCC. The expression level of p21 is decreased with the progression of HCC. Luciferase assays with a luciferase-p21-3′ UTR reporter and its serial deletions identified a 15-bp repressor element at the 3′-UTR of *CDKN1A*, which contains a binding site for *miR-95-3p*. Mutation of the binding site eliminated the regulatory effect of *miR-95-3p* on p21 expression. Posttranscriptional regulation of p21 expression by *miR-95-3p* is mainly on the protein level (suppression of translation). Overexpression of *miR-95-3p* in two different HCC cell lines, HepG2 and SMMC7721, significantly promoted cell proliferation, cell cycle progression and cell migration, whereas a *miR-95-3p* specific inhibitor decreased cell proliferation, cell cycle progression and cell migration. The effects of *miR-95-3p* on cellular functions were rescued by overexpression of p21. Overexpression of *miR-95-3p* promoted cell proliferation and tumor growth in HCC xenograft mouse models. Expression of *miR-95-3p* was significantly higher in HCC samples than in adjacent non-cancerous samples. These results demonstrate that *miR-95-3p* is a potential new marker for HCC and regulates hepatocarcinogenesis by directly targeting *CDKN1A*/p21 expression.

Hepatocellular carcinoma (HCC) is one of the most malignant cancers and causes 662,000 deaths each year worldwide, with more than 50% of deaths occurring in China^1^−^4^. Despite achievements in improved diagnosis and surgical therapy in the past few decades, the prognosis of HCC is poor, and the 5 year survival rate of HCC patients is low^5^. This may be due to incomplete understanding of the molecular pathogenic mechanisms of HCC. Dysregulation of cell cycle progression is a major characteristic of hepatocarcinogenesis, which leads to excessive HCC cell proliferation. Cell cycle is regulated by cyclin-dependent kinases (CDKs), whereas CDK activity is partly inhibited by CDK inhibitors including p21^Waf/Cip1 6^. The p21 is a well-known CDK inhibitor which belongs to the Cip/Kip family of CDK inhibitors and encoded by the *CDKN1A* gene^7^. The p21 protein inhibits the activity of cyclin-CDK2 or -CDK4 complexes and negatively modulates cell cycle progression at phase G1 8. It can also interact with PCNA (proliferating cell nuclear antigen) and is involved in regulation of S phase DNA replication and DNA damage repair^9^,^10^,^11^. As an inhibitor of cell cycle and cell proliferation, p21 plays an important role in tumorigenesis^12^,^13^,^14^.

The expression of *CDKN1A*/p21 is tightly controlled at the transcriptional level primarily by the tumor suppressor protein p53 in response to DNA damage^13^. In addition, the stability of p21 was regulated by multiple mechanisms, including interaction with and phosphorylation by extracellular signal-regulated kinase 2 (ERK2), phosphorylation by c-Jun N-terminal kinase, glycogen synthase kinase (GSK) 3 and AKT^15^,^16^,^17^,^18^. Because of the critical role of p21 in cell cycle and tumor cell proliferation, identification of new regulatory mechanisms for regulation of p21 is important for further understanding of HCC progression and for exploring new treatment and prevention for HCC.

MicroRNAs (miRNAs) are a cluster of noncoding RNA molecules that are approximately 18–25 nucleotides in length and negatively regulate the expression of downstream target genes mainly through direct interaction with the 3′-untranslated regions (3′-UTR) of their corresponding mRNA targets^19^. MiRNAs generally decrease target gene expression through mRNA degradation and/or translational suppression^20^. Increasing evidence in the recent years indicates that many microRNAs play critical roles in tumorigenesis and progression^21^,^22^. In this study, we found that *CDKN1A*/p21 was regulated by *miR-95-3p* by targeting the 3′-UTR. We further showed that expression of *miR-95-3p* was up-regulated in HCC, whereas expression of p21 was decreased with progression of HCC. Overexpression of *miR-95-3p* led to increased tumor cell proliferation and growth in mice. Our study defines *miR-95-3p* as a new oncogenic miRNA involved in hepatocarcinogenesis.

## Results

### Expression of p21 is decreased with progression of HCC

**Figure 1:**
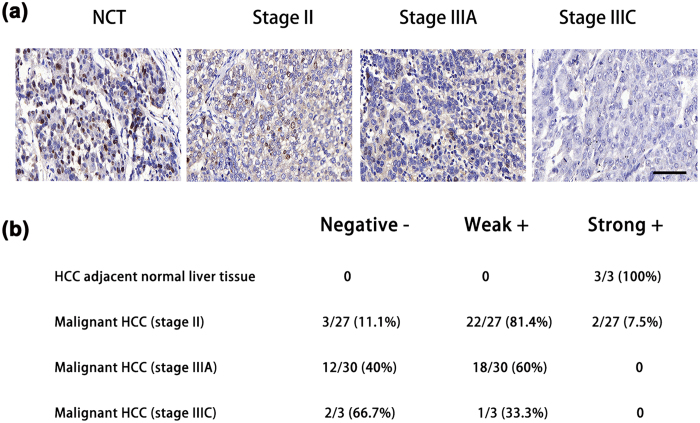
The expression level of p21 was decreased with progression of HCC. **(a**) Representative images of HCC tissue sections at different stages and immunostained with an anti-p21 antibody. NCT: control adjacent non-cancerous tissue; Scale bar = 50 μm. (**b**) Correlation between the intensity of immunostaining signals for p21 and the stage of HCC. Negative, no expression or little expression of p21; weak+, weak expression of p21; strong+, strong expression of p21.

The p21 protein is a tumor suppressor which has been reported to participate in tumor progression and proliferation^6^. In this study, we characterized the expression level of p21 in a total of 60 HCC samples and 3 adjacent non-cancerous tissue samples (NCT) using immunohistochemistry. As shown in Fig. 1, the expression level of p21 was high in the 3 adjacent NCT samples. As HCC progresses from stage II to a higher grade of stage IIIC, the expression levels of p21 were decreased (Fig. 1).

### Identification of a repressor element regulating p21 expression at the 3′-UTR of *CDKN1A*

**Figure 2:**
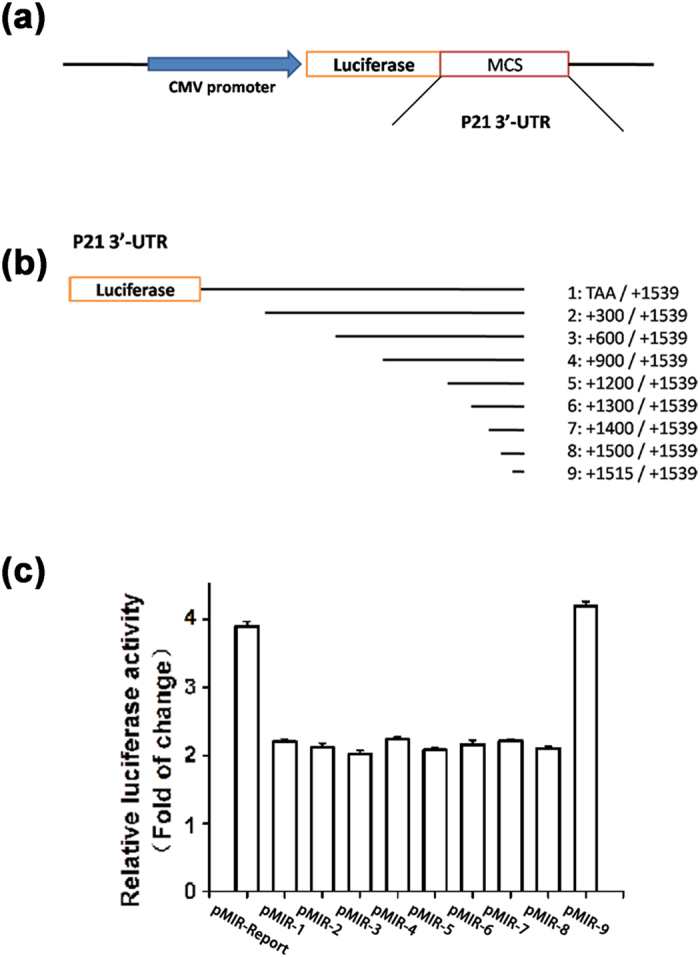
Identification of a critical regulatory element at the 3′-UTR of the *CDKN1A* gene (encoding p21) for regulation of p21 expression. (**a**) Schematic diagram showing a luciferase reporter with the 3′-UTR of the *CDKN1A* gene (a 1,539 bp fragment from the stop codon) sub-cloned after the *luciferase* gene (pMIR-p21-wt or pMIR-1). (**b**) Serial deletions of the 3′-UTR were created in pMIR-1, resulting in pMIR-2 to pMIR-9. (**c**) Luciferase activity for different luciferase reporters. Note that a repressor element was present at the region from +1,500 to +1,515 of the 3′-UTR of the *CDKN1A* gene.

One potential mechanism for down-regulation of p21 in HCC samples is posttranscriptional regulation by microRNA at 3′-UTR of the *CDKN1A* gene (encoding p21). To identify such a posttranscriptional mechanism for p21 regulation, we constructed a luciferase reporter with the 1,539 bp 3′-UTR of *CDKN1A* sub-cloned downstream of the *luciferase* gene in the pMIR-REPORTER vector, resulting in the pMIR-p21-wt reporter (reporter pMIR-1 in Fig. 2a,b). Serial deletions were then made in pMIR-p21-wt, resulting in construction of 8 different deletion mutant reporters (reporter pMIR-2-9 in Fig. 2b). Luciferase assays in HepG2 cells showed that compared to the vector pMIR-REPORTER, luciferase activity of reporter pMIR-1 was reduced, suggesting that there is a repressor element in the 3′-UTR of *CDKN1A* for regulation of *CDKN1A*/p21 expression (Fig. 2c). When the region from +1,500 to +1,515 at the 3′-UTR of *CDKN1A* was deleted, the luciferase activity was significantly increased to the level of pMIR-REPORTER (compare reporter pMIR-9 to others, Fig. 2c). The data suggest that the 3′-UTR repressor element regulating p21 expression is located at the 15-bp region between 1,500 bp and 1,515 bp from the stop codon TAA at the 3′-UTR of *CDKN1A*.

### *MiR-95-3p* regulates expression of p21 by targeting the 3′-UTR of *CDKN1A*

**Figure 3:**
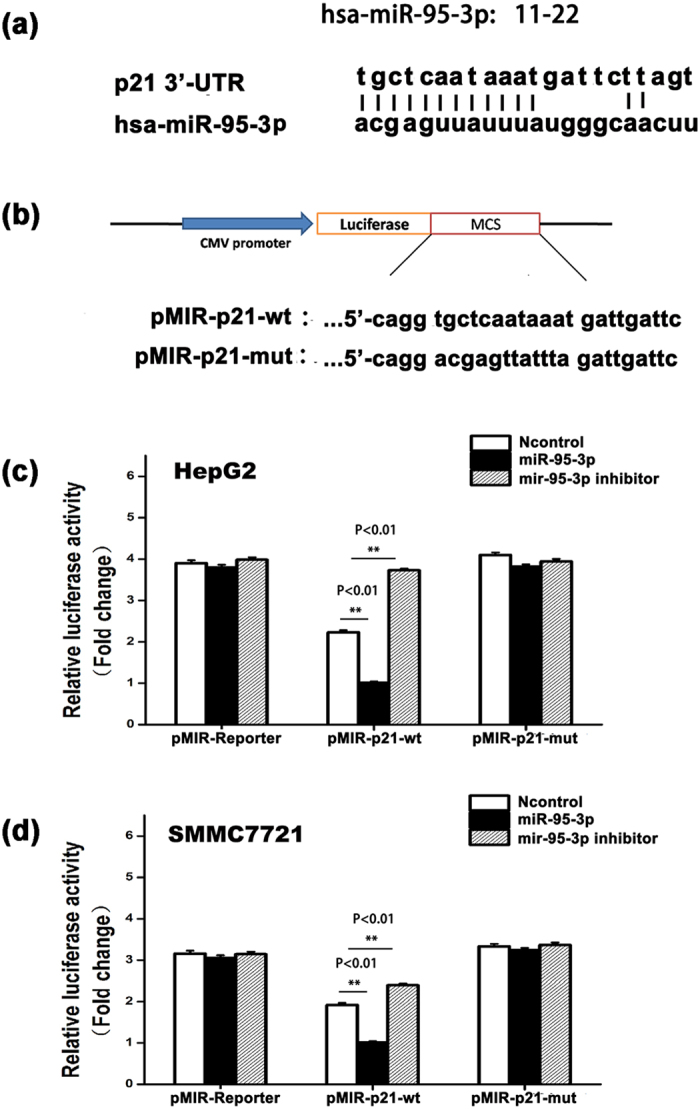
*MiR-95-3p* inhibits p21 expression by directly targeting the 3′-UTR of the *CDKN1A* gene. (**a**) Bioinformatic analysis of the repressor element for the stability of *CDKN1A* mRNA at the region from +1,500 to +1,515 of the 3′-UTR identified a binding site for *miR-95-3p*. (**b**) Schematic diagram showing the wild type (wt) pMIR-p21-wt or mutant pMIR-p21-mut reporter with the *miR-95-3p* binding site mutated. (**c**) Luciferase activity of pMIR-p21-wt or mutant pMIR-p21-mut reporters in the presence of *miR-95-3p* mimics, a negative control miRNA mimics (Ncontrol), and a *miR-95-3p* specific inhibitor in HepG2 cells. (**d**) Similar luciferase assays as in (**c**) but in SMMC7721 cells.

We analyzed the 3′-UTR repressor element regulating p21 expression for a potential microRNA binding site using miRBase (http://www.mirbase.org/). A potential binding site for *Hsa-miR-95-3p* was found at the 3′-UTR repressor element (Fig. 3a).

To determine whether *miR-95-3p* regulates expression of p21, we mutated the *miR-95-3p* binding site in the luciferase reporter pMIR-p21-wt (wild type reporter), resulting in a mutant pMIR-p21-mut reporter (Fig. 3b). The pMIR-p21-wt or pMIR-P21-mut reporter was co-transfected into two different HCC cell lines, HepG2 and SMMC7721 together with *miR-95-3p* mimics, a negative control miRNA mimics (Ncontrol), or a *miR-95-3p* inhibitor and luciferase assays were performed. Compared with empty pMIR-REPORTER vector, the luciferase activity of pMIR-P21-wt was decreased due to endogenous *miR-95-3p*. For wild type reporter pMIR-P21-wt, transfection of *miR-95-3p* mimics significantly reduced the reporter luciferase activity compared to Ncontrol, whereas a *miR-95-3p* specific inhibitor significantly increased the reporter luciferase activity (Fig. 3c,d). However, *miR-95-3p* mimics and the *miR-95-3p* inhibitor did not affect the luciferase activity of the mutant pMIR-p21-mut reporter in which the *miR-95-3p* binding site was mutated (Fig. 3c,d). Together, these data suggest that *miR-95-3p* down-regulates the expression of p21 by directly targeting the 3′-UTR of *CDKN1A*.

**Figure 4:**
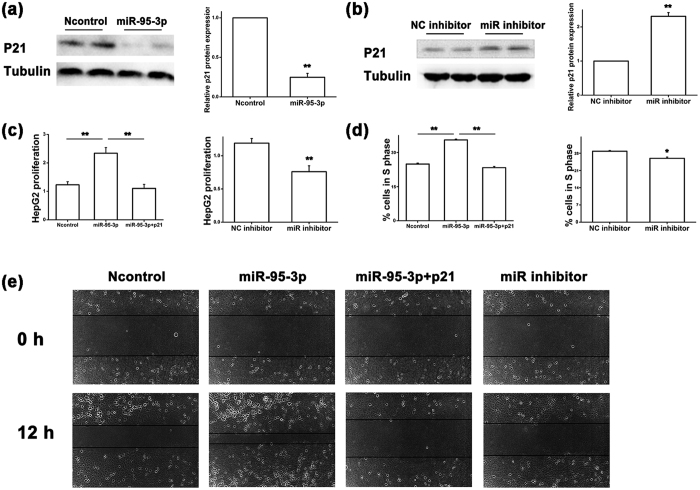
*MiR-95-3p* inhibits expression of p21 at the translational level and promotes HepG2 tumor cell proliferation and migration. (**a**) Western blot analysis for the expression level of the p21 protein in HepG2 cells transfected with *miR-95-3p* mimics compared with negative control miRNA mimics (Ncontrol). The Western blot images are shown at the left, quantified and graphed at the right. β-tubulin was used as a loading control. (**b**) Similar Western blot analysis as in (**a**) but with a *miR-95-3p* inhibitor vs. a negative control miRNA inhibitor (NC inhibitor). (**c**) Proliferation of HepG2 cells transfected with *miR-95-3p* mimics alone or with a p21 expression plasmid vs. Ncontrol mimics, and *miR-95-3p* inhibitor vs. NC inhibitor. (**d**) Percentage of cells at the S phase during cell cycle in HepG2 cells transfected with *miR-95-3p* mimics alone or with a p21 expression plasmid vs. Ncontrol mimics, and *miR-95-3p* inhibitor vs. NC inhibitor. (**e**) Migration of HepG2 cells transfected with *miR-95-3p* mimics alone or with a p21 expression plasmid, Ncontrol mimics, and *miR-95-3p* inhibitor using a wound assay.

**Figure 5:**
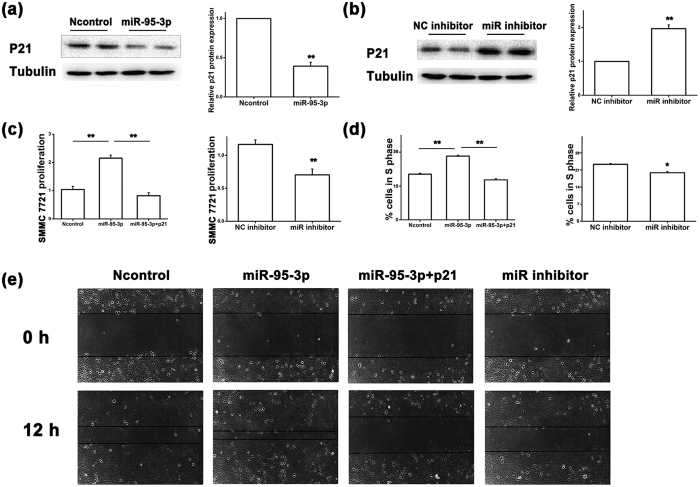
*MiR-95-3p* inhibits expression of p21 at the translational level and promotes SMMC7721 tumor cell proliferation and migration. (**a**) Western blot analysis for the expression level of the p21 protein in SMMC7721 cells transfected with *miR-95-3p* mimics compared with negative control miRNA mimics (Ncontrol). The Western blot images are shown at the left, quantified and graphed at the right. β-tubulin was used as a loading control. (**b**) Similar Western blot analysis as in (**a**) but with a *miR-95-3p* inhibitor vs. a negative control miRNA inhibitor (NC inhibitor). (**c**) Proliferation of SMMC7721 cells transfected with *miR-95-3p* mimics alone or with a p21 expression plasmid vs. Ncontrol mimics, and *miR-95-3p* inhibitor vs. NC inhibitor. (**d**) Percentage of cells at the S phase during cell cycle in SMMC7721 cells transfected with *miR-95-3p* mimics alone or with a p21 expression plasmid vs. Ncontrol mimics, and *miR-95-3p* inhibitor vs. NC inhibitor. (**e**) Migration of SMMC7721 cells transfected with *miR-95-3p* mimics alone or with a p21 expression plasmid, Ncontrol mimics, and *miR-95-3p* inhibitor using a wound assay.

To further validate the finding that *miR-95-3p* regulates the expression of p21, we examined the protein expression level of p21 using Western blot analysis with HepG2 and SMMC7721 cells transfected with *miR-95-3p* mimics vs. Ncontrol, a *miR-95-3p* specific inhibitor, and a negative control miRNA inhibitor (NC inhibitor). As expected, compared to Ncontrol mimics, *miR-95-3p* mimics significantly reduced the protein expression level of p21 (Figs 4a and 5a). On the contrary, the *miR-95-3p* inhibitor significantly increased the protein expression level of p21 in both HepG2 and SMMC7721 cells compared to the NC inhibitor (Figs 4b and 5b).

The expression level of the *CDKN1A* mRNA was also assessed using real time RT-PCR analysis. The real-time RT-PCR analysis showed that the *CDKN1A* mRNA expression level was slightly decreased in HepG2 and SMMC7721 cells transfected with *miR-95-3p* mimics compared to Ncontrol mimics (Supplementary Fig. S1a and Supplementary Fig. S2a), and slightly increased by the *miR-95-3p* inhibitor compared to the NC inhibitor (Supplementary Fig. S1b and Supplementary Fig. S2b). However, the differences were not statistically significant (*P* > 0.05). Taken together, these data suggest that *miR-95-3p* down-regulates p21 expression mainly at the protein or translational level by directly targeting the 3′-UTR.

### *MiR-95-3p* promotes tumor cell proliferation and migration

Considering the finding that *miR-95-3p* negatively regulates expression of tumor suppressor p21, we hypothesized that *miR-95-3p* could promote tumor cell proliferation and migration as well as tumor growth. To determine the impact of *miR-95-3p* on HCC cell proliferation, we transfected HepG2 and SMMC7721 cells with *miR-95-3p* mimics vs. Ncontrol, and the *miR-95-3p* specific inhibitor vs. the NC inhibitor. Cell proliferation assays with the CCK-8 kit revealed that overexpression of *miR-95-3p* significantly promoted cell proliferation of HepG2 and SMMC7721 cells (Figs 4c and 5c). The *miR-95-3p* inhibitor significantly reduced cell proliferation of HepG2 and SMMC7721 cells (Figs 4c and 5c). Furthermore, overexpression of *miR-95-3p* led to an increase of S-phase cells during cell division (Figs 4d and 5d), whereas the *miR-95-3p* inhibitor significantly decreased the number of S-phase cells (Figs 4d and 5d). The wound healing assays showed that overexpression of *miR-95-3p* significantly increased migration of HepG2 and SMMC7721 cells (Figs 4e and 5e), whereas the *miR-95-3p* inhibitor decreased migration of HepG2 and SMMC7721 cells (Figs 4e and 5e).

To examine whether the observed effects of *miR-95-3p* are due to down-regulation of p21, we co-transfected HepG2 and SMMC7721 cells with a p21 expression plasmid together with *miR-95-3p* mimics. Overexpression of p21 significantly abrogated the effects of *miR-95-3p* mimics on cell proliferation, cell division and migration in both HepG2 and SMMC7721 cells (Figs 4c,d,e and 5c,d,e). Taken together, these results suggest that *miR-95-3p* promotes HCC tumor cell proliferation, cell cycle progression and cell migration by directly targeting p21.

### *MiR-95-3p* promotes tumor growth in mice

**Figure 6:**
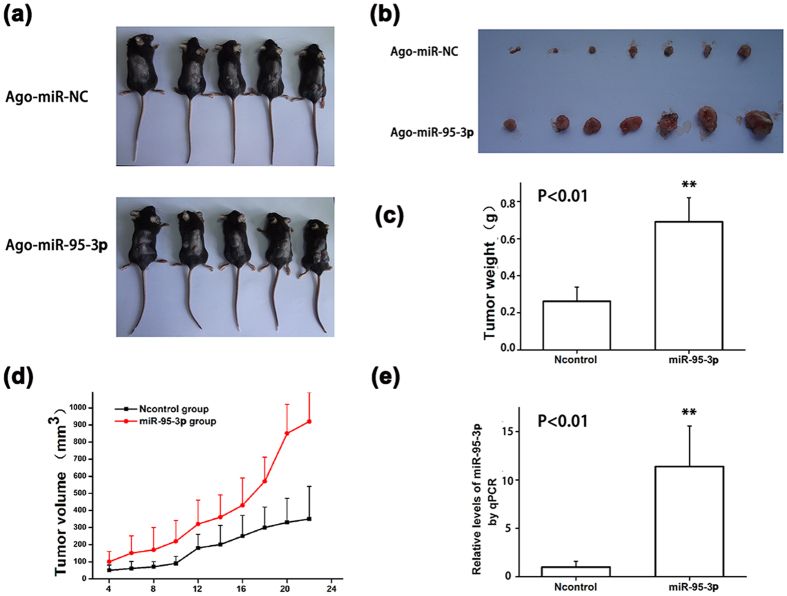
Overexpression of *miR-95-3p* promotes tumor growth in mice. An *in vivo* hepatoma xenograft model was created by subcutaneous injection of 5 × 10^5^ murine hepatoma Hepa1-6 cells transfected with AgomiR-95-3p or AgomiR-NC in mice. (**a**) Tumor growth for the AgomiR-95-3p group and the AgomiR-NC group. (**b**) Photos of tumors excised from mice on experimental day 22. (**c**) Tumor weight for the AgomiR-95-3p group and the AgomiR-NC group. (**d**) Tumor growth curves for the AgomiR-95-3p group and the AgomiR-NC group. The tumor volume (V) was computed using a formula of V = (L × W^2^) × 0.5 (n = 7/group). (**e**) Quantitative qPCR analysis showing that the expression level of *miR-95-3p* was significantly increased in tumors isolated from the AgomiR-95-3p group compared with the AgomiR-NC group.

We used mouse Hepa1-6 cells to create a xenograft tumor model in mice for HCC. AgomiR-95-3P or AgomiR-NC was transfected into Hepa1-6 cells. Transfected cells were injected subcutaneously into the back of the C57BL/6 mice and tumor growth was monitored. At the end of the study, tumors were excised, weighed and photographed (Fig. 6a). The tumor size from the AgomiR-95-3p group was larger than those from the AgomiR-NC group (Fig. 6a,b). The tumor weight from the AgomiR-95-3p group was significantly heavier than that from the AgomiR-NC group (Fig. 6c). The tumor growth curves showed that the tumors from the AgomiR-95-3p group grew faster than those from the AgomiR-NC control group (Fig. 6d). Real time qPCR analysis showed that the expression level of *miR-95-3p* was significantly higher in tumors from the AgomiR-95-3p group than those from the AgomiR-NC group (Fig. 6e). Together, these data suggest that overexpression of *miR-95-3p* promoted tumor growth *in vivo*.

**Figure 7:**
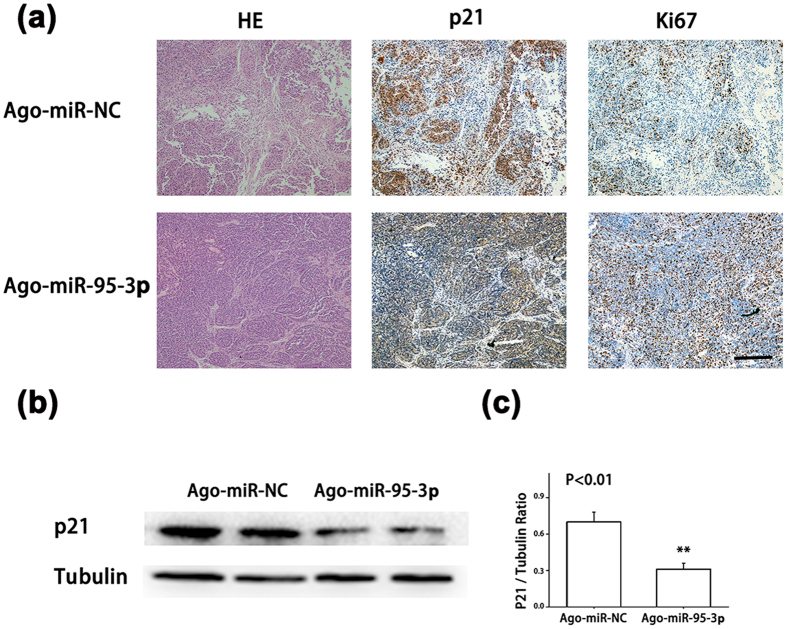
Overexpression of *miR-95-3p* decreases p21 expression and promotes tumor cell proliferation in mice. An *in vivo* hepatoma xenograft model was established as in Fig. 6. (**a**) Representative images of tumor sections from the AgomiR-95-3p group and the AgomiR-NC group. H&E, haematoxylin and eosin stain; p21, immunostaining with an anti-p21 antibody; Ki67, immunostaining with an anti-Ki67 antibody; Scale bar = 100 μm. (**b**) Western blot analysis for p21. β-tubulin was used as an internal control. (**c**) The p21/tubulin ratio in (**b**) was quantified.

We used immunohistochemical staining with an anti-Ki67 antibody to examine the density of positive Ki67 proliferating cells. An anti-p21 antibody was also used to examine the expression level of p21 in mouse tumor sections. The density of positive Ki67 cells was much higher in tumors from the AgomiR-95-3p group than those from the AgomiR-NC group (Fig. 7a). Immunohistochemical analysis showed that the expression level of p21 was much lower in tumors from the AgomiR-95-3p group than those from the AgomiR-NC group (Fig. 7a). Western blot analysis also showed that p21 expression was lower in the AgomiR-95-3p group than in the AgomiR-NC group (Fig. 7b,c). These data provide *in vivo* evidence that *miR-95-3p* down-regulates the expression of p21, which leads to increased cell proliferation and tumor growth.

### *MiR-95-3p* expression was increased in HCC tissues

**Figure 8:**
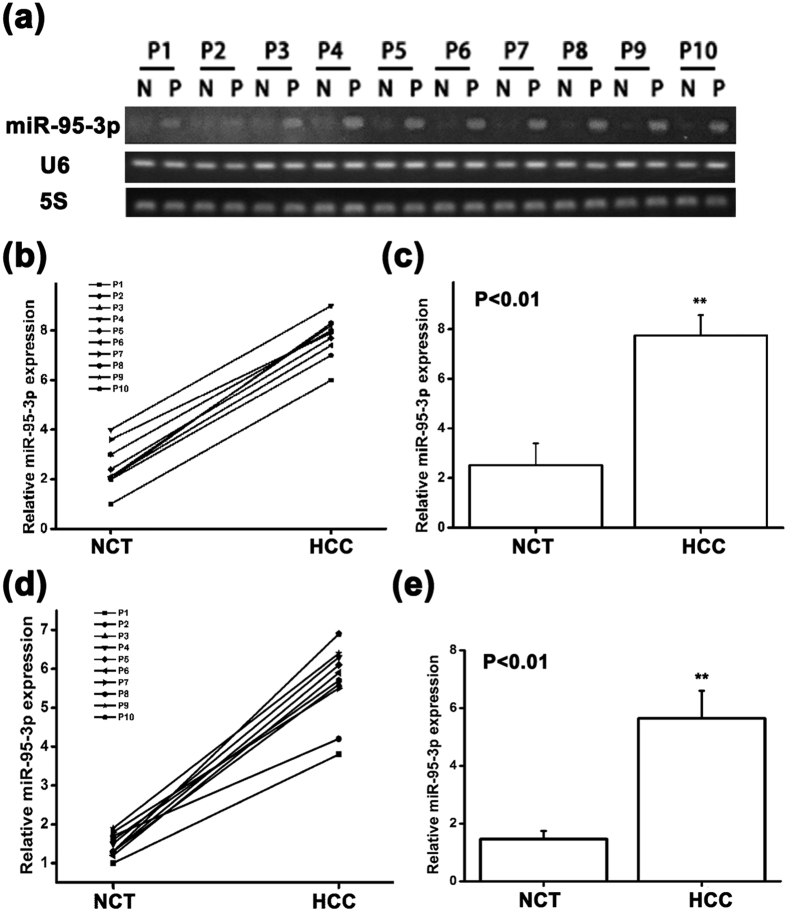
The expression level of *miR-95-3p* is higher in HCC tissues than in normal adjacent non-cancerous tissues. (**a**) Semi-quantitative RT-PCR analysis showed a higher expression level of *miR-95-3p* in HCC tissue samples than in normal adjacent tissue samples. (**b**) PCR bands in (**a**) were quantified and plotted. The ratio of the expression level of *miR-95-3p* over *U6 RNA* in HCC tissue sample was higher than that in the normal adjacent non-cancerous tissue sample in all 10 groups. (**c**) The relative *miR-95-3p* expression level over *U6 RNA* in human HCC tissues was significantly higher than that in normal adjacent non-cancerous tissues. (**d**) PCR bands in (**a**) were quantified and plotted. The ratio of the expression level of *miR-95-3p* over *5S RNA* in HCC tissue sample was higher than that in the normal adjacent non-cancerous tissue sample in all 10 groups. (**e**) The relative *miR-95-3p* expression level over *5S RNA* in human HCC tissues was significantly higher than that in normal adjacent non-cancerous tissues.

We used semi-quantitative RT-PCR analysis to measure the relative expression level of *miR-95-3p* in 10 pairs of HCC samples and their respective adjacent non-cancerous samples. In each pair, the expression level of *miR-95-3p* was consistently higher in HCC samples than in adjacent non-cancerous samples (Fig. 8a,b,d). Together, the expression level of *miR-95-3p* was significantly higher in HCC samples than in adjacent non-cancerous samples (*P* < 0.01; Fig. 8c,e).

## Discussion

In this study, we demonstrated that the expression level of *miR-95-3p* was consistently up-regulated in HCC tissues compared with adjacent non-cancerous tissues in all 10 groups of samples examined (Fig. 8). Moreover, the expression levels of *miR-95-3p* in HCC tissues were higher than that from all non-tumorous tissues (Fig. 8). Because the sample size is small, future studies with large sample sizes are needed to further validate this interesting finding. If confirmed, *miR-95-3p* may serve as a potential marker for diagnosis of HCC.

We found that overexpression of *miR-95-3p* significantly promoted HepG2 and SMMC7721 cell proliferation and migration in cultured cells (Figs 4 and 5) and Hepa1-6 tumor cell proliferation and tumor growth in mice (Figs 6 and 7). Therefore, it is highly likely that up-regulation of *miR-95-3p* in HCC tissues is causative to hepatocarcinogenesis and tumor growth. Mechanistically, we showed that *miR-95-3p* causes hepatocarcinogenesis by posttranscriptional suppression of p21 expression by binding to the 3′-UTR. Several pieces of evidence strongly supports the conclusion. First, we found that the expression level of p21 is reduced with the progression of HCC. When HCC reached stageIIIC, there is little expression of p21 (Fig. 1). Second, we constructed several luciferase reporters which contain different regions of 3′-UTR of *CDKN1A* cloned downstream of the *luciferase* gene. Luciferase assays showed that there is a potential repressor element in the 3′-UTR of *CDKN1A*, which contains a miRNA binding site for *miR-95-3p* (Fig. 2). Overexpression of *miR-95-3p* mimics decreased the expression of p21, and the effect was eliminated by mutation of the binding site for *miR-95-3p* (Figs 3, 4 and 5). Moreover, a *miR-95-3p* specific inhibitor increased the expression of p21 (Figs 4 and 5). Third, overexpression of *miR-95-3p* in a mouse hepatoma xenograft model decreased expression of p21 (Fig. 7). Together, we conclude that *CDKN1A* encoding p21 is a downstream gene regulated by *miR-95-3p*.

Similar to our finding of up-regulation of *miR-95-3p* in HCC tissues and promotion of tumorigenesis by overexpression of *miR-95-3p*, three other reports revealed involvement of *miR-95-3p* in other types of tumors. The expression level of *miR-95-3p* was reported to be up-regulated in glioma tissues and down-regulation of *miR-95-3p* inhibited proliferation and invasion and promoted apoptosis of glioma cells by targeting *CELF2* encoding CUGBP- and ETR-3-like family 2 23. The expression level of *miR-95-3p* was also up-regulated in human prostate and breast cancer tissues or after ionizing radiation. Overexpression of *miR-95-3p* promoted radiation resistance and cell proliferation following ionizing radiation and increased tumor cell invasiveness as well as tumor growth by targeting the sphingolipid phosphatase SGPP1^24^. The expression level of *miR-95-3p* was up-regulated in human non-small cell lung cancer tissues and overexpression of *miR-95-3p* increased tumor growth in xenograft mouse models by targeting *SNX1* encoding sorting nexin1^25^. Our study identified the *CDKN1A* gene encoding p21 as a new target gene for *miR-95-3p.* In contrast, overexpression of *miR-95-3p* was found to inhibit brain metastasis of lung adenocarcinoma by suppressing expression of cyclin D1^26^. Together, these studies indicate that *miR-95-3p* plays important roles in tumorigenesis in different types of cancer by targeting different downstream genes.

There are several limitations with the present study. (1) We have shown that down-regulation of p21 appears to be responsible for the effect of *miR-95-3p* on cell proliferation, cell cycle progression and cell migration in two independent HCC cell lines because co-expression of p21 rescued the effects of *miR-95-3p* (Figs 4 and 5). However, as discussed above, *miR-95-3p* also regulates other target genes such as *CELF2, SGPP1, SNX1* and many other unidentified target genes, future studies are needed to investigate the roles of other *miR-95-3p* target genes in the pathogenesis of HCC. (2) There are other microRNAs that can also regulate the expression of p21. It should be interesting to investigate the roles of other p21-regulating *miRNAs* in the pathogenesis of HCC. (3) Overexpression of microRNAs may have off-target effects, and caution should be used in interpreting the data, although complementary studies of both *miR-95-3p* mimics and a *miR-95-3p* specific inhibitor strengthened the conclusions. (4) The 15-bp 3′-UTR region missing in pMIR-9 (Fig. 2) also contains a less-well matched seed sequence for *miR-545-5p* (6-nucleotide match). It may be interesting to examine whether *miR-545-5p* also regulates expression of *CDKN1A* in the future. (5) The sample sizes of three normal healthy liver specimens for the immunohistochemistry of p21 (Fig. 1) and 10 pairs of HCC tissue samples and adjacent non-cancerous samples for semi-quantitative RT-PCR analysis of *miR-95-3p* (Fig. 8) are small. Future studies with large sample sizes are needed to further validate the findings of decreased p21 expression and increased *miR-95-3p* expression in HCC.

In summary, we have found that *miR-95-3p* is up-regulated in HCC tissues compared with adjacent non-cancerous tissues. Overexpression of *miR-95-3p* promoted tumor cell proliferation and migration in cultured cells and tumor growth in xenograft mouse models through negative posttranscriptional regulation of p21 by directly targeting the 3′-UTR. This study establishes *miR-95-3p* as a potential biomarker for diagnosis of HCC and as a new therapeutic target for treatment and prevention of HCC.

## Methods

### Cell lines and human tumor samples

Three HCC cell lines, including HepG2, SMMC7721, and Hepa1-6, were cultured in DMEM media supplemented with 10% fetal bovine serum (FBS) (Gibco Life Technologies, Gaitherburg, MD, USA) under 5% CO_2_ and at 37 °C. We screened HepG2 and SMMC7721 cells for mutations in genes encoding p53, p21 and MDM2, but no mutation was found.

HCC tissues and matched human adjacent non-cancerous liver tissue samples (NCT) were collected from patients undergoing surgical resection of tumors in Affiliated Hospitals of Huazhong University of Science and Technology. Clinical stages were classified according to the International Union against Cancer TNM classification system^27^. The demographical features of age and sex and clinical stages of HCC patients were listed in Supplementary Tables S1 and S2. This study was approved by the Ethics Committees on human subject research of Huazhong University of Science and Technology and local institutions and written informed consent was obtained from all study subjects. This study conformed to guidelines set forth by the Declaration of Helsinki.

### Construction of plasmids

The whole 3′-UTR of the *CDKN1A* gene encoding p21 (1,539 bp) was amplified by polymerase chain reaction (PCR) analysis with human genomic DNA as the template. The PCR product was cut with *Spe I* and *Hind III* and cloned into the pMIR-REPORT luciferase vector (Promega, Madison, WI, USA), resulting in a luciferase reporter referred to as pMIR-p21-wt. Serial deletions were created in pMIR-p21-wt (pMIR-1), resulting in different deletion mutants: pMIR-p21-f-300bp (pMIR-2), pMIR-p21-f-600bp (pMIR-3), pMIR-p21-f-900bp (pMIR-4), pMIR-p21-f-1200bp (pMIR-5), pMIR-p21-f-1300bp (pMIR-6), pMIR-p21-f-1400bp (pMIR-7), pMIR-p21-f-1500bp (pMIR-8), and pMIR-p21-f-1515bp (pMIR-9). In all reporters, the 3′-UTR fragment of *CDKN1A* was cloned downstream of the firefly *luciferase* coding region, which is under the control of the *CMV* promoter.

The *miR-95-3p* binding site at the 3′-UTR of *CDKN1A* was mutated in the pMIR-p21-wt reporter using site-directed mutagenesis by PCR as previously described^28^,^29^,^30^,^31^, resulting in reporter pMIR-p21-mut.

Primers used in this study for plasmid construction and mutagenesis were listed in Supplementary Table S3.

### MicroRNA reagents

We purchased *miR-95-3p* mimics, negative control miRNA mimics (Ncontrol), a *miR-95-3p* inhibitor, a negative control *miR-95-3p* inhibitor (NC inhibitor), AgomiR-95-3p and control AgomiR-NC from Guangzhou RioboBio (Guangzhou, Guangdong, China).

### Dual luciferase assays

Luciferase assays were performed as described previously by us^32^,^33^,^34^. HepG2 and SMMC7721 cells were plated in a 24-well plate and co-transfected with 200 ng of either pMIR-p21-wt, pMIR-p21-mut, or a pMIR-p21-deletion mutants together with 100 nM of *miR-95-3p* mimics, negative control mimics (NControl) or a *miR-95-3p* specific inhibitor as well as 10 ng of the pRL-TK vector containing the renilla *luciferase* gene (Promega, Madison, WI, USA) using Lipofectamine 2000 (Gibco Life Technologies, Gaithersburg, MD, USA). Luciferase assays were carried out using the Dual-Glo luciferase assay kit according to the manufacturer’s instruction (Promega, Madison, WI, USA). The final luciferase activity was expressed as the firefly activity over the renilla luciferase activity.

### Measurement of miRNA expression using quantitative RT-PCR (qRT-PCR) analysis

We quantified the relative expression level of *miR-95-3p* using stem-loop real time PCR as previously described^35^,^36^.

### Real-time RT-PCR analysis

Total cell or tissue RNA samples were extracted and reverse-transcribed to cDNA by the RevertAid First Strand cDNA synthesis kit (Fermentas) using random primers (Promega, Madison, WI, USA). The expression level of *CDKN1A* mRNA was quantified using a FastStart Universal SYBR Green Master kit (Roche Applied Science, Mannheim, Germany) as described by us previously^31^,^32^,^33^,^37^,^38^. The endogenous control was *ACTB* (encoding β-actin). Primers for real-time RT- PCR analysis were listed in Supplementary Table S3. Data were analyzed using the 2^−△△Ct^ method as described^36^.

### Western blot analysis

Western blot analysis was carried out as described by us previously^31^,^33^. The antibodies used in this study include an anti-p21 antibody (1:500 dilution, Proteintech, Wuhan, China) and an anti-β-tubulin antibody (1:2000 dilution, Proteintech, Wuhan, China).

### *In vivo* tumor growth assays

Both animal care and experimental procedures were approved by the Ethics Committee on Animal Research of College of Life Science and Technology of Huazhong University of Science and Technology and performed according to the Guidelines for the Care and Use of Animals for Research by the Ministry of Science and Technology of the P. R. China. C57BL/6 mice (males, 7-week-old) were purchased from Center for Medical Experimental Animals of Wuhan University (Wuhan, Hubei, China).

*In vivo* tumor growth assays were performed as previously described^39^,^40^. In brief, 150 nM of AgomiR-95-3p or AgomiR-NC was transfected into mouse Hepa1-6 cells using Lipofectamin RNAiMAX (Gibco Life Technologies, Gaithersburg, MD, USA). After 24 h of transfection, cells were harvested, washed with cold PBS and suspended at a concentration of 5 × 10^6^ cells/ml in PBS. C57BL/6 mice were divided into 2 groups (n = 7). These mice were subcutaneously injected with 5 × 10^5^ Hepa1-6 cells (100 μl) on the back. Tumor growth was examined every 2 days beginning at day 4. Tumor length (L) and width (W) were measured and the formula of V = (L × W^2^) × 0.5 was used to calculate the tumor volume (V).

At the end of the experiment at day 22, the tumors were excised from mice and weighed.

### Immunohistochemical staining

Tumors excised from mice were fixed in 4% paraformaldehyde and used for immunohistochemical staining. Immunohistochemical staining was performed as described^31^,^33^,^41^. Briefly, 4.5 μm-tumor sections were immunostained with an antibody against p21 (1:100 dilution, Proteintech, Wuhan, China) and an antibody against Ki-67 (1:100 dilution, Proteintech, Wuhan, China). DAB PI was used for staining of nuclei. Hematoxylin-eosin staining was used to evaluate the morphology of the tumor sections as described by us^31^. Images were captured under a microscope.

### Cell cycle assay

Cell cycle analysis was performed as described^31^ with cells transfected with *miR-95-3p* mimics, negative control miRNA mimics (Ncontrol), a *miR-95-3p* inhibitor or a negative control inhibitor (NC inhibitor). Cells were stained with propidium iodide (PI). Cell cycle analysis was performed using a Beckman Coulter Cytomics FC 500 flow cytometry and CXP software (Beckman Coulter).

### Cell proliferation and migration assays

Cells were seeded onto 96-well plates, and assayed for proliferation at 48 h using a CCK-8 kit (Dojindo Laboratories, Kumamoto, Japan) according to the manufacturer’s instruction. Cell proliferation was analyzed by measurement of absorbance at 450 nm using a microplate reader as described^42^.

For cell migration, we used a wound assay as described^34^. Cells (5 × 10^5^) were seeded onto six-well plates and cultured under standard conditions. When the cell density reached confluence, a wound was made by scraping the cell monolayer with a 200 μl pipette tip. Cell migration was determined by measuring the movement of cells into the scraped area. The process of wound closure was monitored and photographed 12 hours after wounding under a microscope.

### Bioinformatic and statistical analyses

We used the miRBase (http://www.mirbase.org/) database to predict the putative binding sites of miRNAs.

All quantitative data for statistical analyses were from at least three independent experiments. The data were presented as means ± SD (standard deviation). Statistical analysis was performed using a Student’s t test. A *P* value of <0.05 was considered to be statistically significant.

## Additional Information

**How to cite this article**: Ye, J. *et al.* Up-regulation of *miR-95-3p* in hepatocellular carcinoma promotes tumorigenesis by targeting p21 expression. *Sci. Rep.* **6**, 34034; doi: 10.1038/srep34034 (2016).

## Supplementary Material

Supplementary Information
