# Supplementary material for: Up-regulation of miR-95-3p in hepatocellular carcinoma promotes tumorigenesis by targeting p21 expression
Source: Sci Rep. 2016 Oct 4;6:34034. doi: 10.1038/srep34034 (PMC5048429; doi:10.1038/srep34034)
Supplement: Supplementary Information [file srep34034-s1.doc]

**Supplementary information**

**Up-regulation of *miR-95-3p* in hepatocellular carcinoma promotes tumorigenesis by targeting p21 expression**

**Jian Ye^1,*^, Yufeng Yao^1,*^, Qixue Song^1,*^, Sisi Li^1,*^, Zhenkun Hu^1^, Yubing Yu^1^, Changqing Hu^1^, Xingwen Da^1^, Hui Li^1^, Qiuyun Chen^2^, Qing K. Wang^1,2,*^**

^1^Key Laboratory of Molecular Biophysics of the Ministry of Education, College of Life Science and Technology and Center for Human Genome Research，Huazhong University of Science and Technology, Wuhan, P. R. China. ^2^Center for Cardiovascular Genetics, Department of Molecular Cardiology, Cleveland Clinic; Department of Molecular Medicine/CCLCM, Department of Genetics and Genome Sciences, Case Western Reserve University, Cleveland, OH 44195, USA. ^*^These authors contributed equally to this work. Corresponding author. Tel.: +86 27 87793502; fax: +86 27 87793502. Email addresses: [qkwang@hust.edu.cn](mailto:qkwang@hust.edu.cn)/wangq2@ccf.org (Q. K. Wang)

**Supplementary Table S1**

**Demographical features of HCC patients used for analysis of p21 expression by immunohistochemistry.**

| Patient ID | Age (year) | Sex | Pathological Diagnosis | Tumor TNM Staging |
| --- | --- | --- | --- | --- |
| 1 | 38 | male | Hepatocellular Carcinoma | T3N0M0 |
| 2 | 38 | male | Hepatocellular Carcinoma | T3N0M0 |
| 3 | 38 | male | Hepatocellular Carcinoma | T3N0M0 |
| 4 | 63 | Female | Hepatocellular Carcinoma | T2N0M0 |
| 5 | 63 | Female | Hepatocellular Carcinoma | T2N0M0 |
| 6 | 63 | Female | Hepatocellular Carcinoma | T2N0M0 |
| 7 | 71 | male | Hepatocellular Carcinoma | T3N0M0 |
| 8 | 71 | male | Hepatocellular Carcinoma | T3N0M0 |
| 9 | 71 | male | Hepatocellular Carcinoma | T3N0M0 |
| 10 | 50 | male | Hepatocellular Carcinoma | T3N0M0 |
| 11 | 50 | male | Hepatocellular Carcinoma | T3N0M0 |
| 12 | 50 | male | Hepatocellular Carcinoma | T3N0M0 |
| 13 | 40 | male | Hepatocellular Carcinoma | T2N0M0 |
| 14 | 40 | male | Hepatocellular Carcinoma | T2N0M0 |
| 15 | 40 | male | Hepatocellular Carcinoma | T2N0M0 |
| 16 | 50 | male | Hepatocellular Carcinoma | T2N0M0 |
| 17 | 50 | male | Hepatocellular Carcinoma | T2N0M0 |
| 18 | 50 | male | Hepatocellular Carcinoma | T2N0M0 |
| 19 | 78 | male | Hepatocellular Carcinoma | T2N0M0 |
| 20 | 78 | male | Hepatocellular Carcinoma | T2N0M0 |
| 21 | 78 | male | Hepatocellular Carcinoma | T2N0M0 |
| 22 | 35 | male | Hepatocellular Carcinoma | T3N0M0 |
| 23 | 35 | male | Hepatocellular Carcinoma | T3N0M0 |
| 24 | 35 | male | Hepatocellular Carcinoma | T3N0M0 |
| 25 | 43 | Female | Hepatocellular Carcinoma | T3N0M0 |
| 26 | 43 | Female | Hepatocellular Carcinoma | T3N0M0 |
| 27 | 43 | Female | Hepatocellular Carcinoma | T3N0M0 |
| 28 | 36 | male | Hepatocellular Carcinoma | T2N0M0 |
| 29 | 36 | male | Hepatocellular Carcinoma | T2N0M0 |
| 30 | 36 | male | Hepatocellular Carcinoma | T2N0M0 |
| 31 | 52 | male | Hepatocellular Carcinoma | T3N1M0 |
| 32 | 52 | male | Hepatocellular Carcinoma | T3N1M0 |
| 33 | 52 | male | Hepatocellular Carcinoma | T3N1M0 |
| 34 | 52 | male | Hepatocellular Carcinoma | T2N0M0 |
| 35 | 52 | male | Hepatocellular Carcinoma | T2N0M0 |
| 36 | 52 | male | Hepatocellular Carcinoma | T2N0M0 |
| 37 | 48 | male | Hepatocellular Carcinoma | T2N0M0 |
| 38 | 48 | male | Hepatocellular Carcinoma | T2N0M0 |
| 39 | 48 | male | Hepatocellular Carcinoma | T2N0M0 |
| 40 | 35 | male | Hepatocellular Carcinoma | T2N0M0 |
| 41 | 35 | male | Hepatocellular Carcinoma | T2N0M0 |
| 42 | 35 | male | Hepatocellular Carcinoma | T2N0M0 |
| 43 | 50 | male | Hepatocellular Carcinoma | T3N0M0 |
| 44 | 50 | male | Hepatocellular Carcinoma | T3N0M0 |
| 45 | 50 | male | Hepatocellular Carcinoma | T3N0M0 |
| 46 | 56 | male | Hepatocellular Carcinoma | T3N0M0 |
| 47 | 56 | male | Hepatocellular Carcinoma | T3N0M0 |
| 48 | 56 | male | Hepatocellular Carcinoma | T3N0M0 |
| 49 | 43 | male | Hepatocellular Carcinoma | T3N0M0 |
| 50 | 43 | male | Hepatocellular Carcinoma | T3N0M0 |
| 51 | 43 | male | Hepatocellular Carcinoma | T3N0M0 |
| 52 | 48 | male | Hepatocellular Carcinoma | T3N0M0 |
| 53 | 48 | male | Hepatocellular Carcinoma | T3N0M0 |
| 54 | 48 | male | Hepatocellular Carcinoma | T3N0M0 |
| 55 | 63 | male | Hepatocellular Carcinoma | T3N0M0 |
| 56 | 63 | male | Hepatocellular Carcinoma | T3N0M0 |
| 57 | 63 | male | Hepatocellular Carcinoma | T3N0M0 |
| 58 | 57 | male | Hepatocellular Carcinoma | T2N0M0 |
| 59 | 57 | male | Hepatocellular Carcinoma | T2N0M0 |
| 60 | 57 | male | Hepatocellular Carcinoma | T2N0M0 |

**Supplementary Table S2**

Demographical features of HCC patients used for analysis of *miR-95-3p* expression by semi-quantitative RT-PCR analysis.

| Patient ID | Age (year) | Sex | Pathological Diagnosis | Tumor TNM Staging |
| --- | --- | --- | --- | --- |
| 1 | 54 | male | Hepatocellular Carcinoma | T3N0M0 |
| 2 | 54 | male | Hepatocellular Carcinoma | T3N0M0 |
| 3 | 55 | male | Hepatocellular Carcinoma | T3N0M0 |
| 4 | 48 | Female | Hepatocellular Carcinoma | T3N0M0 |
| 5 | 57 | Female | Hepatocellular Carcinoma | T3N0M0 |
| 6 | 38 | Female | Hepatocellular Carcinoma | T3N0M0 |
| 7 | 41 | male | Hepatocellular Carcinoma | T3N0M0 |
| 8 | 52 | male | Hepatocellular Carcinoma | T3N0M0 |
| 9 | 61 | male | Hepatocellular Carcinoma | T3N0M0 |
| 10 | 53 | male | Hepatocellular Carcinoma | T3N0M0 |

**Supplementary Table S3**

Sequences of primers used in this study.

| **Primer** | **Sequence** |
| --- | --- |
| Primers for construction of pMIR-p21-wt-3’-UTR(pMIR-1) | F: atgcactagttccgcccacaggaagcctg  R: atgcaagcttttacaagtaaagtcactaagaatcatttattgagca |
| Primers for construction of pMIR-2-3’UTR(pMIR-2) | F: atgcactagtgtgggccggcttcatgcc  R: atgcaagcttttacaagtaaagtcactaagaatcatttattgagca |
| Primers for construction of pMIR-3-3’UTR(pMIR-3) | F: atgcactagttggctcttgatacccccctctgt  R: atgcaagcttttacaagtaaagtcactaagaatcatttattgagca |
| Primers for construction of pMIR-4-3’UTR(pMIR-4) | F: atgcactagtactggaaggggaagggacacaca  R: atgcaagcttttacaagtaaagtcactaagaatcatttattgagca |
| Primers for construction of pMIR-5-3’UTR(pMIR-5) | F: atgcactagttagaggctatggacagggcatgc  R: atgcaagcttttacaagtaaagtcactaagaatcatttattgagca |
| Primers for construction of pMIR-6-3’UTR(pMIR-6) | F: atgcactagttagtgtacttggagtattggggtctgacc  R: atgcaagcttttacaagtaaagtcactaagaatcatttattgagca |
| Primers for construction of pMIR-7-3’UTR(pMIR-7) | F: atgcactagtcccgtttctccacctagactgtaaacc  R: atgcaagcttttacaagtaaagtcactaagaatcatttattgagca |
| Primers for construction of pMIR-8-3’UTR(pMIR-8) | atgcactagtcaggtgctcaataaatgattcttagtgactttacttgtaa aagcttcgta |
| Primers for construction of pMIR-9-3’UTR(pMIR-9) | atgcactagttgattcttagtgactttacttgtaaaagcttcgta |
| Primers for construction of  pMIR-p21-mut-3’-UTR | F: atgcactagttccgcccacaggaagcctg  R: atgcaagcttttacaagtaaagtcactaagaatctaaataactcgtc |
| *CDKN1A* real time primer (for mouse) | F: gctggagggcaacttcgtctg  R: caggccgctcagacaccaga |
| *CDKN1A* real time primer (for human) | F: gcgaggccgggatgagttg  R: ccggcgtttggagtggtagaa |
| *ACTB* real time primer (for mouse) | F: cgtccacccgcgagcacag  R: cgacgaccagcgcagcgatat |
| *ACTB* real time primer (for human) | F: cgcgagaagatgacccagat  R: tcaccggagtccatcacgat |
| *U6* RT primer | aacgcttcacgaatttgcgt |
| *miR-95-3p* RT primer | ctcaactggtgtcgtggagtcggcaattcagttgagtgctcaat |
| *U6* real time primer | F: ctcgcttcggcagcaca  R: aacgcttcacgaatttgcgt |
| *miR-95-3p* real time primer | F: acactccagctgggttcaacgggtatttat  R: tggtgtcgtggagtcg |

**
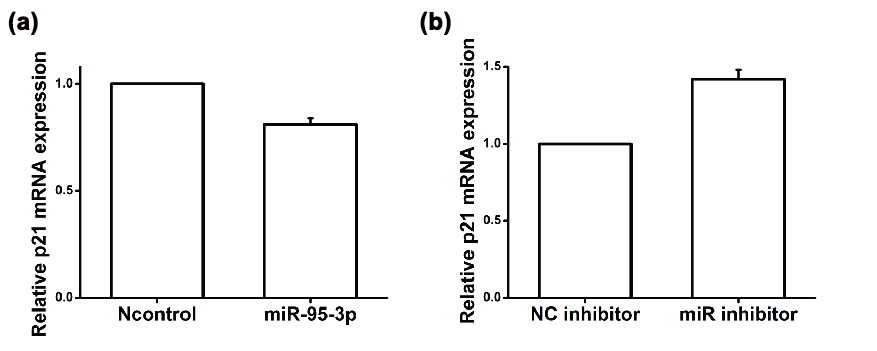
**

**Supplementary Figure S1. Quantitative RT-PCR analysis showing the expression level of the *CDKN1A* mRNA in HepG2 cells transfected with *miR-95-3p* mimics, negative control miRNA mimics (Ncontrol), *miR-95-3p* inhibitor, and negative control miRNA inhibitor (NC inhibitor).** (**a**) Quantitative RT-PCR data for the expression level of the *CDKN1A* mRNA in HepG2 cells transfected with *miR-95-3p* mimics and Ncontrol (*P*>0.05). (**b**) Quantitative RT-PCR data for the expression level of the *CDKN1A* mRNA in HepG2 cells transfected with *miR-95-3p* inhibitor and NC inhibitor (*P*>0.05).

**
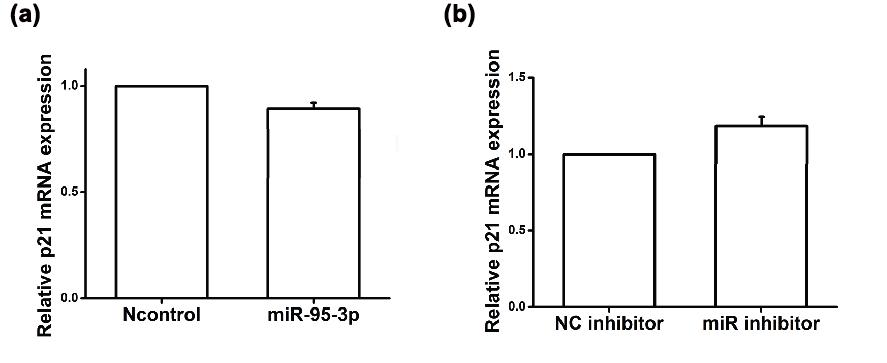
**

**Supplementary Figure S2. Quantitative RT-PCR analysis showing the expression level of the *CDKN1A* mRNA in SMMC7721 cells transfected with *miR-95-3p* mimics, negative control miRNA mimics (Ncontrol), *miR-95-3p* inhibitor, and negative control miRNA inhibitor (NC inhibitor).** (**a**) Quantitative RT-PCR data for the expression level of the *CDKN1A* mRNA in SMMC7721 cells transfected with *miR-95-3p* mimics and Ncontrol (*P*>0.05). (**b**) Quantitative RT-PCR data for the expression level of the *CDKN1A* mRNA in SMMC7721 cells transfected with *miR-95-3p* inhibitor and NC inhibitor (*P*>0.05).
